# COVID-19 in the Middle East and North Africa region: an urgent call for reliable, disaggregated and openly shared data

**DOI:** 10.1136/bmjgh-2021-005175

**Published:** 2021-02-09

**Authors:** Sarah Wehbe, Sasha A. Fahme, Anthony Rizk, Ghina R. Mumtaz, Jocelyn DeJong, Abla M. Sibai

**Affiliations:** 1 Faculty of Medicine, American University of Beirut, Beirut, Lebanon; 2 Faculty of Health Sciences, American University of Beirut, Beirut, Lebanon; 3 Graduate Institute of International and Development Studies, Geneva, Switzerland

**Keywords:** epidemiology, health systems evaluation, public health

**Summary box**

- In contrast to other regions, most Arab countries in the Middle East and North Africa (MENA) region do not publicly report comprehensive and disaggregated epidemiological data on COVID-19.
- The COVID-19 pandemic exemplifies long-standing underinvestment and undervaluation of routine sources of data, a paucity of available disaggregated data and challenges to data sharing across several countries of the MENA region, notably those that are long stricken by conflicts and displacement.
- The COVID-19 pandemic should serve as an impetus for more comprehensive, robust, disaggregated and publicly available evidence, and this can be addressed through prioritised governmental expenditure and use of available digital technologies.
- Comprehensive and reliable data are essential in understanding the implications of the health crises, generating meaningful epidemiological research and developing prompt and contextualised responses.

As of December 2020, Arab countries of the Middle East and North Africa (MENA) region have reported more than 3.2 million confirmed cases of SARS-CoV-2 and 55 000 deaths from COVID-19.^1^ The Institute for Health Metrics and Evaluation noted a threefold increase in the number of deaths in the region between September and December 2020, with COVID-19 projected to become the fourth leading cause of death by early 2021.^2^ Yet significant discrepancies in both indicators and quality of data reported across the MENA region limit our understanding of the scope and the implications of the pandemic in the Arab context. The MENA region is distinctly conflict-affected and displacement-affected, which may foster unique vulnerabilities to SARS-CoV-2 transmission and illness severity. Low testing rates, limited data on excess mortality and poor vital registration systems, which are further weakened in the context of chronic political unrest, all contribute to consistent under-reporting in the region.

In order to develop a timely and context-informed response to the pandemic and more recently to the vaccination statistics, publicly available and disaggregated data are critical to identify health needs and interpret the impact of the region’s competing crises of protracted wars, forced displacement and economic decline on already fragile health systems and poor resource availability. At present in the MENA region, as elsewhere, data sources for COVID-19 cases have primarily been limited to government reporting of daily aggregates of incidence, morbidity and mortality counts.^3^ There are, however, notable yet limited exceptions,^4 5^ with some Arab countries sharing more granular data. Table 1 summarises the findings of an extensive review of the publicly available data from governmental (ministerial websites including social media and WHO-EMRO country dashboards) and non-governmental (public dashboards) sources. Online supplemental file 1 provides the full lists of data sources and indicates which countries make disaggregated data publicly available according to a number of pertinent stratifiers.

10.1136/bmjgh-2021-005175.supp1 Supplementary data

**Table 1:** Publicly available disaggregated COVID-19 data among Arab countries by key selected stratifiers

	Sex		Age		Subnational region		Refugee status		Comorbid conditions*	Hospital admission
	Incidence	Mortality	Incidence	Mortality	Incidence	Mortality	Incidence	Mortality		
Algeria	X	X	X	X	X	X				X
Bahrain	X	X		X						X
Comoros										
Djibouti	X								X	
Egypt										
Iraq	X	X	X	X	X	X	X	X	X	X
Jordan	X	X	X	X	X					X
Kuwait										X
Lebanon	X	X	X	X	X	X			X	X
Libya					X	X				
Mauritania	X		X		X	X				
Morocco	X	X			X	X				X
Oman					X	X				X
Palestine	X	X	X	X	X	X				X
Qatar	X	X	X	X					X	X
Saudi Arabia	X				X	X				X
Somalia	X		X	X	X	X				X
Sudan	X		X		X	X				X
Syria					X	X				X
Tunisia	X	X	X	X	X	X				X
United Arab Emirates										
Yemen	X	X	X	X	X	X				X
Total (22)	15	10	11	10	15	14	1	1	4	16

*Comorbid conditions as reported among deceased and not COVID-19 cases.

Sex-disaggregated data on COVID-19 incidence and mortality, respectively, have been reported in only 15 and 10 out of the 22 Arab countries. The significance of COVID-19-related gender disparities has been recognised worldwide^6^ and carries far-reaching implications in the Arab context, which has the world’s lowest female labour force participation rates.^7^ Additionally, gendered caregiving responsibilities, which include caring for ill relatives, may place Arab women at greater risk of contracting the virus. Sex-disaggregated data are thus vital for developing prompt and contextualised responses.

Age-stratified COVID-19 mortality estimates are reported in only 10 Arab countries, and information on existing comorbidities among deceased individuals is specified only in four. Additionally, only 68% of countries provide data on SARS-CoV-2 incidence by geographical distribution. These findings are in stark contrast to initiatives in other regions worldwide, where subnational age-stratified and sex-stratified data on COVID-19 incidence, hospitalisation and mortality are routinely reported, such as in Canada, China, Europe and Latin America.^8 9^ Some local health departments in the USA like that of New York City—the former epicentre of the pandemic—also report incidence and mortality data stratified by poverty level.^10^

Similarly, although the MENA region is home to the world’s largest population of forcibly displaced persons, data on COVID-19 morbidity and mortality among internally displaced and refugee populations are lacking in all countries except for Iraq.^5^ This paucity of regional data on refugees is not unique to the pandemic. Indeed, the lack of integrated monitoring systems, the scarcity of disaggregated data on risk factors, incidence and mortality among displaced populations and refugees in camps and in host communities, as well as the suboptimal sharing and use of primary data by service organisations are only some of the obstacles to achieving an effective and evidence-informed response to this humanitarian crisis.^11^ Consequently, health policy decisions pertaining to refugees are often not data-driven, which is particularly dangerous during a pandemic.

The region-wide scarcity of robust, disaggregated and publicly available evidence predates the pandemic. It reflects historical underinvestment in necessary infrastructure and near-total absence of open-access data repositories, in addition to a lack of data governance and regulatory mechanisms, notably in resource-scarce settings. Routine sources of data that are drivers of research and policy making, such as death certification, epidemiological surveillance units and de-identified patient records from hospitals and healthcare centres, are either unavailable or inaccessible for primary or secondary analysis. This has been apparent in prior epidemics as well. For instance, HIV publicly available data and research in the MENA region still lag substantially behind the global literature, even as the number of HIV infections in the region has increased—a scarcity that has often led to misrepresentation of the situation and poor outbreak management and response.^12^ Additionally, and in autocratic settings in particular, the transparency of public health data may be perceived by governments as a political or even security liability that could potentially undermine the legitimacy of authoritarian leadership.

There needs to be a paradigm shift in how public officials conceive health-related data in the MENA region. Governments need to advance simple and decisive changes that enhance data availability and accessibility, starting by addressing the region’s COVID-19 knowledge gap. These may include training of civil servants on data de-identification and strengthening collaborations between the public sector and academic institutions. Additionally, task forces need to be designated to create or expand on visually engaging open-access dashboards that provide detailed testing, incidence, hospitalisation and mortality data, stratified by key demographic characteristics, such as gender, age, geographical location, refugee status and comorbidities, among others.^13^ Initiatives can follow the example of the WHO-EMRO Dynamic Infographic Dashboard for Iraq, a comprehensive and interactive tool with detailed disaggregated data across geographically diverse regions and populations.^5^ As demonstrated by this platform and others in the region, including a similar tool developed in Palestine,^14^ the technology needed to create such open data systems is readily available and should be broadly adapted.^15^ The rise of digital technologies and reporting through social media platforms provides key opportunities to moving forward, which will require stronger political commitment and the requisite financial support.^16^ Public health professionals can also draw on lessons learnt from other humanitarian crises in the region, such as the August 2020 Beirut explosion or the protracted forced displacement of Palestinians, both of which have inspired interactive open-access mapping initiatives.^17 18^

The COVID-19 pandemic calls for strong and prioritised public investment in data infrastructure in the MENA region—a push forward that balances public health needs with concerns for data privacy and confidentiality. The COVID-19 ‘infodemic’, likely made worse in the MENA by decades of distrust in government leadership and public institutions,^19^ poses significant health risks, particularly amid the current new variants and waves of the virus as well as large-scale vaccination campaigns. This misinformation can only be addressed with unified, transparent public health messaging informed by accurate and reliable data. More than ever, solidarity and integrative approaches are needed to build regional and national health data systems that would outlast the current pandemic. COVID-19 provides such an imperative.
