# Supplementary material for: COVID-19 in the Middle East and North Africa region: an urgent call for reliable, disaggregated and openly shared data
Source: BMJ Glob Health. 2021 Feb 9;6(2):e005175. doi: 10.1136/bmjgh-2021-005175 (PMC7874901; doi:10.1136/bmjgh-2021-005175)
Supplement: Supplementary data [file bmjgh-2021-005175supp001.pdf]

Supplementary file:

Availability and Sources of disaggregated COVID-19-related data among Arab countries by key selected stratifiers*

Incidence by Sex (Available in 15 countries, not available in 7 countries)	
Algeria	https://covid19.cdta.dz/dashboard/production/index.php<br>https://datastudio.google.com/u/0/reporting/ebfabb34-4a74-4822-a2bc-516cfab21fd2/page/yHrIB
Bahrain	https://globalhealth5050.org/the-sex-gender-and-covid-19-project/the-data-tracker/?explore=country&country=Bahrain#search
Djibouti	https://globalhealth5050.org/the-sex-gender-and-covid-19-project/the-data-%20tracker/?explore=country&country=djibouti#search
Iraq	https://app.powerbi.com/view?r=eyJrIjoiNjljMDhiYmItZTlhMS00MDlhLTg3MjItMDNmM2FhNzE5NmM4IiwidCI6ImY2MTBjMGI3LWJkMjQtNGIzOS04MTBiLTNkYzI4MGFmYjU5MCIsImMiOjh9
Jordan	https://corona.moh.gov.jo/ar
Lebanon	https://www.moph.gov.lb/en/Media
Mauritania	https://www.sante.gov.mr/?p=3569
Morocco	https://globalhealth5050.org/the-sex-gender-and-covid-19-project/the-data-tracker/?explore=country&country=morocco#search
Palestine	https://app.powerbi.com/view?r=eyJrIjoiODJlYWM1YTEtNDAxZS00OTFlLThkZjktNDA1ODY2OGQ3NGJkIiwidCI6ImY2MTBjMGI3LWJkMjQtNGIzOS04MTBiLTNkYzI4MGFmYjU5MCIsImMiOjh9<br>http://site.moh.ps/index/covid19/LanguageVersion/1/Language/ar
Qatar	https://globalhealth5050.org/the-sex-gender-and-covid-19-project/the-data-tracker/?explore=country&country=qatar#search
Saudi Arabia	https://www.moh.gov.sa/en/Ministry/MediaCenter/News/Pages/default.aspx
Somalia	https://bmgf.maps.arcgis.com/apps/opsdashboard/index.html#/d0d9a939c5fa401caa3a7447e72b2017
Sudan	http://www.fmoh.gov.sd
Tunisia	http://www.onmne.tn/category/actualites/<br>https://globalhealth5050.org/the-sex-gender-and-covid-19-project/the-data-tracker/?explore=country&country=tunisia#search
Yemen	https://app.powerbi.com/view?r=eyJrIjoiZjE2NzJjZDItNDgyZi00NDFkLWFlMjItNjA2MjIwMWYzODJkIiwidCI6ImY2MTBjMGI3LWJkMjQtNGIzOS04MTBiLTNkYzI4MGFmYjU5MCIsImMiOjh9

**Mortality by Sex (Available in 10 countries, not available in 12 countries)**	
Algeria	https://datastudio.google.com/u/0/reporting/ebfabb34-4a74-4822-a2bc-516cfab21fd2/page/yHrIB
Bahrain	https://twitter.com/moh_bahrain?lang=ar
Iraq	https://app.powerbi.com/view?r=eyJrIjoiNjljMDhiYmItZTlhMS00MDlhLTg3MjItMDNmM2FhNzE5NmM4IiwidCI6ImY2MTBjMGI3LWJkMjQtNGIzOS04MTBiLTNkYzI4MGFmYjU5MCIsImMiOjh9 (both)
Jordan	https://corona.moh.gov.jo/ar
Lebanon	https://www.moph.gov.lb/en/Media
Morocco	https://globalhealth5050.org/the-sex-gender-and-covid-19-project/the-data-tracker/?explore=country&country=morocco#search
Palestine	http://site.moh.ps/index/covid19/LanguageVersion/1/Language/ar
Qatar	https://www.medrxiv.org/content/10.1101/2020.07.16.20155317v2.full.pdf
Tunisia	https://globalhealth5050.org/the-sex-gender-and-covid-19-project/the-data-tracker/?explore=country&country=tunisia#search
Yemen	https://app.powerbi.com/view?r=eyJrIjoiZjE2NzJjZDItNDgyZi00NDFkLWFlMjItNjA2MjIwMWYzODJkIiwidCI6ImY2MTBjMGI3LWJkMjQtNGIzOS04MTBiLTNkYzI4MGFmYjU5MCIsImMiOjh9

**Incidence by Age (Available in 11 countries, not available in 11 countries)**	
Algeria	https://covid19.cdta.dz/dashboard/production/index.php
Iraq	https://app.powerbi.com/view?r=eyJrIjoiNjljMDhiYmItZTlhMS00MDlhLTg3MjItMDNmM2FhNzE5NmM4IiwidCI6ImY2MTBjMGI3LWJkMjQtNGIzOS04MTBiLTNkYzI4MGFmYjU5MCIsImMiOjh9
Jordan	https://corona.moh.gov.jo/ar
Lebanon	https://www.moph.gov.lb/en/Media
Mauritania	https://www.sante.gov.mr/?p=3569
Palestine	https://app.powerbi.com/view?r=eyJrIjoiODJlYWM1YTEtNDAxZS00OTFlLThkZjktNDA1ODY2OGQ3NGJkIiwidCI6ImY2MTBjMGI3LWJkMjQtNGIzOS04MTBiLTNkYzI4MGFmYjU5MCIsImMiOjh9 http://site.moh.ps/index/covid19/LanguageVersion/1/Language/ar
Qatar	https://www.medrxiv.org/content/10.1101/2020.07.16.20155317v2.full.pdf
Somalia	https://bmgf.maps.arcgis.com/apps/opsdashboard/index.html#/d0d9a939c5fa401caa3a7447e72b2017
Sudan	http://www.fmoh.gov.sd
Tunisia	http://www.onmne.tn/category/actualites/
Yemen	https://app.powerbi.com/view?r=eyJrIjoiZjE2NzJjZDItNDgyZi00NDFkLWFlMjItNjA2MjIwMWYzODJkIiwidCI6ImY2MTBjMGI3LWJkMjQtNGIzOS04MTBiLTNkYzI4MGFmYjU5MCIsImMiOjh9

**Mortality by Age (Available in 10 countries, not available in 12 countries)**	
Algeria	https://datastudio.google.com/u/0/reporting/ebfabb34-4a74-4822-a2bc-516cfab21fd2/page/yHrIB
Bahrain	https://twitter.com/moh_bahrain?lang=ar
Iraq	https://app.powerbi.com/view?r=eyJrIjoiNjljMDhiYmItZTlhMS00MDlhLTg3MjItMDNmM2FhNzE5NmM4IiwidCI6ImY2MTBjMGI3LWJkMjQtNGIzOS04MTBiLTNkYzI4MGFmYjU5MCIsImMiOjh9
Jordan	https://corona.moh.gov.jo/ar
Lebanon	https://www.moph.gov.lb/en/Media
Palestine	http://site.moh.ps/index/covid19/LanguageVersion/1/Language/ar
Qatar	https://www.medrxiv.org/content/10.1101/2020.07.16.20155317v2.full.pdf
Somalia	https://bmgf.maps.arcgis.com/apps/opsdashboard/index.html#/d0d9a939c5fa401caa3a7447e72b2017
Tunisia	http://www.onmne.tn/category/actualites/
Yemen	https://app.powerbi.com/view?r=eyJrIjoiZjE2NzJjZDItNDgyZi00NDFkLWFlMjItNjA2MjIwMWYzODJkIiwidCI6ImY2MTBjMGI3LWJkMjQtNGIzOS04MTBiLTNkYzI4MGFmYjU5MCIsImMiOjh9

**Incidence by Subnational Region (Available in 15 countries, not available in 7 countries)**	
Algeria	https://covid19.cdta.dz/dashboard/production/index.php<br>https://twitter.com/Sante_Gouv_dz<br>https://datastudio.google.com/u/0/reporting/ebfabb34-4a74-4822-a2bc-516cfab21fd2/page/yHrIB
Iraq	https://app.powerbi.com/view?r=eyJrIjoiNjljMDhiYmItZTlhMS00MDlhLTg3MjItMDNmM2FhNzE5NmM4IiwidCI6ImY2MTBjMGI3LWJkMjQtNGIzOS04MTBiLTNkYzI4MGFmYjU5MCIsImMiOjh9
Jordan	https://corona.moh.gov.jo/ar
Lebanon	https://www.moph.gov.lb/en/Media
Libya	https://ncdc.org.ly/Ar/situation-of-corona/
Mauritania	https://www.sante.gov.mr/?p=3569
Morocco	https://www.sante.gov.ma/Pages/activites.aspx?activiteID=329
Oman	https://covid19.moh.gov.om/#/home
Palestine	https://app.powerbi.com/view?r=eyJrIjoiODJlYWM1YTEtNDAxZS00OTFlLThkZjktNDA1ODY2OGQ3NGJkIiwidCI6ImY2MTBjMGI3LWJkMjQtNGIzOS04MTBiLTNkYzI4MGFmYjU5MCIsImMiOjh9<br>http://site.moh.ps/index/covid19/LanguageVersion/1/Language/ar
Saudi Arabia	https://covid19.moh.gov.sa
Somalia	https://bmgf.maps.arcgis.com/apps/opsdashboard/index.html#/d0d9a939c5fa401caa3a7447e72b2017
Sudan	http://www.fmoh.gov.sd<br>https://www.facebook.com/FMOH.SUDAN/
Syria	https://app.powerbi.com/view?r=eyJrIjoiNTA0NWMxZmYtMDJiMC00ZWU0LTllNTktZTViZjYwYThjZmUzIiwidCI6ImY2MTBjMGI3LWJkMjQtNGIzOS04MTBiLTNkYzI4MGFmYjU5MCIsImMiOjh9
Tunisia	http://www.onmne.tn/category/actualites/<br>https://ageos-tunisie.maps.arcgis.com/apps/opsdashboard/index.html#/f0ca1c1bfc67492b8f14dba1f24331f1
Yemen	https://app.powerbi.com/view?r=eyJrIjoiZjE2NzJjZDItNDgyZi00NDFkLWFlMjItNjA2MjIwMWYzODJkIiwidCI6ImY2MTBjMGI3LWJkMjQtNGIzOS04MTBiLTNkYzI4MGFmYjU5MCIsImMiOjh9

**Mortality by Subnational Region (Available in 14 countries, not available in 8 countries)**	
Algeria	https://datastudio.google.com/u/0/reporting/ebfabb34-4a74-4822-a2bc-516cfab21fd2/page/yHrIB
Iraq	https://app.powerbi.com/view?r=eyJrIjoiNjljMDhiYmItZTlhMS00MDlhLTg3MjItMDNmM2FhNzE5NmM4IiwidCI6ImY2MTBjMGI3LWJkMjQtNGIzOS04MTBiLTNkYzI4MGFmYjU5MCIsImMiOjh9
Lebanon	https://www.moph.gov.lb/en/Media
Libya	https://ncdc.org.ly/Ar/situation-of-corona/
Mauritania	https://www.sante.gov.mr/?p=3569
Morocco	https://www.sante.gov.ma/Pages/activites.aspx?activiteID=329
Oman	https://covid19.moh.gov.om/#/home
Palestine	http://site.moh.ps/index/covid19/LanguageVersion/1/Language/ar
Saudi Arabia	https://covid19.moh.gov.sa/
Somalia	https://bmgf.maps.arcgis.com/apps/opsdashboard/index.html#/d0d9a939c5fa401caa3a7447e72b2017
Sudan	http://www.fmoh.gov.sd<br>https://www.facebook.com/FMOH.SUDAN/
Syria	https://app.powerbi.com/view?r=eyJrIjoiNTA0NWMxZmYtMDJiMC00ZWU0LTllNTktZTViZjYwYThjZmUzIiwidCI6ImY2MTBjMGI3LWJkMjQtNGIzOS04MTBiLTNkYzI4MGFmYjU5MCIsImMiOjh9
Tunisia	http://www.onmne.tn/category/actualites/<br>https://ageos-tunisie.maps.arcgis.com/apps/opsdashboard/index.html#/f0ca1c1bfc67492b8f14dba1f24331f1
Yemen	https://app.powerbi.com/view?r=eyJrIjoiZjE2NzJjZDItNDgyZi00NDFkLWFlMjItNjA2MjIwMWYzODJkIiwidCI6ImY2MTBjMGI3LWJkMjQtNGIzOS04MTBiLTNkYzI4MGFmYjU5MCIsImMiOjh9

**Incidence and Mortality among Refugees (Available in 1 country, not available in 21 countries)**	
Iraq	https://app.powerbi.com/view?r=eyJrIjoiNjljMDhiYmItZTlhMS00MDlhLTg3MjItMDNmM2FhNzE5NmM4IiwidCI6ImY2MTBjMGI3LWJkMjQtNGIzOS04MTBiLTNkYzI4MGFmYjU5MCIsImMiOjh9

**Comorbid conditions reported amongst deceased (Available in 4 countries, not available in 18 countries)**	
Djibouti	https://twitter.com/MinSantedj
Iraq	https://app.powerbi.com/view?r=eyJrIjoiNjljMDhiYmItZTlhMS00MDlhLTg3MjItMDNmM2FhNzE5NmM4IiwidCI6ImY2MTBjMGI3LWJkMjQtNGIzOS04MTBiLTNkYzI4MGFmYjU5MCIsImMiOjh9
Lebanon	https://www.moph.gov.lb/en/Media
Qatar	https://www.medrxiv.org/content/10.1101/2020.07.15.20154211v2.full.pdf

**Hospital admission (Available in 16 countries, not available in 6 countries)**	
Algeria	https://twitter.com/Sante_Gouv_dz
Bahrain	https://twitter.com/moh_bahrain?lang=ar
Iraq	https://app.powerbi.com/view?r=eyJrIjoiNjljMDhiYmItZTlhMS00MDlhLTg3MjItMDNmM2FhNzE5NmM4IiwidCI6ImY2MTBjMGI3LWJkMjQtNGIzOS04MTBiLTNkYzI4MGFmYjU5MCIsImMiOjh9
Jordan	https://corona.moh.gov.jo/ar
Kuwait	https://corona.e.gov.kw/En<br>https://twitter.com/KUWAIT_MOH
Lebanon	https://www.moph.gov.lb/en/Media
Morocco	https://www.sante.gov.ma/Pages/activites.aspx?activiteID=329
Oman	https://twitter.com/OmanVSCovid19
Palestine	https://app.powerbi.com/view?r=eyJrIjoiODJlYWM1YTEtNDAxZS00OTFlLThkZjktNDA1ODY2OGQ3NGJkIiwidCI6ImY2MTBjMGI3LWJkMjQtNGIzOS04MTBiLTNkYzI4MGFmYjU5MCIsImMiOjh9<br>http://site.moh.ps/index/covid19/LanguageVersion/1/Language/ar
Qatar	https://covid19.moph.gov.qa/EN/Pages/default.aspx
Saudi Arabia	https://covid19.moh.gov.sa/<br>https://www.moh.gov.sa/en/Ministry/MediaCenter/News/Pages/default.aspx
Somalia	https://bmgf.maps.arcgis.com/apps/opsdashboard/index.html#/d0d9a939c5fa401caa3a7447e72b2017
Sudan	http://www.fmoh.gov.sd
Syria	https://app.powerbi.com/view?r=eyJrIjoiNTA0NWMxZmYtMDJiMC00ZWU0LTllNTktZTViZjYwYThjZmUzIiwidCI6ImY2MTBjMGI3LWJkMjQtNGIzOS04MTBiLTNkYzI4MGFmYjU5MCIsImMiOjh9
Tunisia	http://www.onmne.tn/category/actualites/
Yemen	https://app.powerbi.com/view?r=eyJrIjoiZjE2NzJjZDItNDgyZi00NDFkLWFlMjItNjA2MjIwMWYzODJkIiwidCI6ImY2MTBjMGI3LWJkMjQtNGIzOS04MTBiLTNkYzI4MGFmYjU5MCIsImMiOjh9

* The lack of a uniform source of data for some countries and consistently updated data for others necessitated the search drawing on various resources, including ministry websites, social media, and public dashboards.
